# Association between severe acute malnutrition during childhood and blood pressure during adulthood in the eastern Democratic Republic of the Congo: the Lwiro cohort study

**DOI:** 10.1186/s12889-021-10908-4

**Published:** 2021-05-02

**Authors:** Pacifique Mwene-Batu, Daniel Lemogoum, Laurène de le Hoye, Ghislain Bisimwa, Michel P. Hermans, Jimmy Minani, Gaylord Amani, Guy-Quesney Mateso, Justin Cirhuza Cikomola, Michelle Dramaix, Philippe Donnen

**Affiliations:** 1 Ecole Régionale de Santé Publique, Université Catholique de Bukavu, Bukavu, Democratic Republic of Congo; 2 Ecole de Santé Publique, Université Libre de Bruxelles, Brussels, Belgium; 3 Nutritional department, Centre de Recherche en Sciences Naturelles, Lwiro, Democratic Republic of Congo; 4 Institute of Health and Society, Université Catholique de Louvain, Brussels, Belgium; 5 Hôpital Provincial General de Reference de Bukavu, Université Catholique de Bukavu, Bukavu, Democratic Republic of Congo; 6 Hôpital ULB-Erasme , Université Libre de Bruxelles, Brussels, Belgium; 7 Division of Endocrinology & Nutrition, Cliniques universitaires St-Luc, Université Catholique de Louvain, Brussels, Belgium; 8 Faculty of Medicine, Univeristé catholique de Bukavu, Bukavu, Democratic Republic of Congo

**Keywords:** Long-term, Childhood acute malnutrition, Hypertension, Follow-up, DR Congo

## Abstract

**Introduction:**

Little is known about the long-term outcomes of Severe Acute Malnutrition (SAM) during childhood. As such, this study aims to explore the association between childhood SAM and blood pressure (BP) in adulthood in a context without nutrition transition.

**Methodology:**

We identified 524 adults (Median age: 22 years) who were treated for SAM during childhood in Eastern DRC between 1988 and 2007. They were compared with 407 age-and-sex matched subjects with no history of SAM in the community. The variables examined for this study were the systolic (SBP), diastolic (DBP), mean (MBP) blood pressure (BP) and pulse pressure (PP), as well as high blood pressure (HBP) defined as BP ≥ 140/90 mmHg and/or use of BP-lowering drug(s) in adulthood. For comparison, linear and logistic regression models were used for analysing continuous and dichotomous variables, respectively.

**Results:**

Of the 524 exposed located, 145 were selected according to age. A total of 97 unexposed were recruited. Compared to unexposed, exposed had slightly higher SBP and PP after adjusting for occupation, body mass index (BMI) and food consumption [SBP = 1.4 mmHg (− 2.2, 4.8) and PP = 2.6 mmHg (− 0.3, 6.0)]. However, their DBP was lower than that of the unexposed [− 1.6 mmHg (− 4.6, 1.5)]. MBP and creatinine levels were similar between the two groups. The prevalence of HBP adjusted for age was higher among exposed than unexposed (9.7% vs 5.3%). In addition, the odds of having HBP was higher among exposed than unexposed, however the observed difference was not statistically significant [Odds Ratio (OR) 1.9 (0.7, 5.6)]. Finally, using multiple regression analysis, although the effect was not significant, SAM was a major contributor to HBP [adjusted OR 3.1 (0.9,10.9), *p* = 0.064], while only male gender and higher BMI (overweight/obesity) emerged as independent predictors of HBP among this young study population.

**Conclusions:**

This study suggests that an episode of SAM in childhood has a weak impact on BP variability in young Congolese adults (from DRC) living in an environment without nutrition transition. However, people who experienced a period of SAM tended to have a higher prevalence of HBP and a much higher risk of developing HBP than unexposed. Additional multicentre studies involving a larger cohort would provide greater understanding of the impact of SAM on the overall risk of BP disorders during adulthood.

## Introduction

Acute malnutrition (AM) is a major public health problem in low-income countries (LICs) [1]. Every year, more than 5.9 million children under the age of five die worldwide and 45% of those deaths are attributed to malnutrition [2]. There are currently 17 million children across the world suffering from severe acute malnutrition (SAM), of whom 27% live in Africa [1].

In the Democratic Republic of the Congo (DRC), malnutrition is still a major public health problem, with a chronic malnutrition prevalence of 43% among children under the age of five [3]. In South Kivu, a province located in the east of the country, malnutrition has been endemic since the 1960s [4]. According to the report of the National Nutrition Program (PRONANUT) published in 2019, one in two children under the age of 5 has chronic malnutrition (CM) and 3% have AM [5]. In this province, a recent study identified an increased prevalence of HBP and obesity [6].

According to the developmental origin theory for non-communicable chronic diseases (NCDs), there is a causal association between undernutrition during childhood and NCDs in adulthood [7]. This phenomenon, known as the ‘Developmental origins of health and disease’ is well-documented today [7]. It was especially studied in high- and middle-income countries (HMICs) [7–11].

Despite growing evidence on the negative long-term effects of childhood undernutrition observed in HMICs, there is surprisingly little data on the long-term outcomes of children treated for SAM in LICs [12–14].

Studies conducted in Uganda and Malawi (LICs) showed that catch-up growth after an episode of SAM or delayed childhood growth, respectively, were associated with increased blood pressure (BP) in adolescence [14], and that pre-pubescent survivors of childhood SAM were at greater risk of subsequent NCDs, even though no clinical or biological marker of these subsequent morbidities was identified seven years after nutritional rehabilitation [13]. Still in Africa, a study conducted in Nigeria showed a higher prevalence of HBP in participants born during the Nigerian Civil War (1968–1970), and who had been exposed to severe famine in utero and/or during childhood compared to unexposed subjects who lived in the same region before the war, in 1965–1967 [15].

However, the presumed role of childhood malnutrition in the increased burden of NCDs and related risk factors in the DRC has not been explored.

This was the reason for conducting this study, which aimed to assess the association between childhood SAM (before the age of five years) and BP in a cohort of young adults screened 11 to 30 years after nutritional rehabilitation, with no nutrition transition (environment with a monotonous, undiversified and low-quality food situation), living in the east of the DRC.

## Methodology

### Study area

The study was conducted at the “Centre de Recherche en Sciences Naturelles de Lwiro (CRSNLwiro)”, in the health zones (HZ) of Katana and Miti-Murhesa in South Kivu, in the DRC. The Nutrition Department of this center has one pediatric hospital and four integrated health centers which monitor the health and nutrition of children in the community [4].

### Population studied

The size of the sample was determined by the number of patients admitted for SAM to the Lwiro pediatric hospital (HPL) from 1988 to 2007 and living in Miti-Murhesa and Katana in 2018 [16]. A total of 1981 patients were treated for SAM during childhood at the HPL between 1988 and 2007. On admission to this hospital, the median age was 41 months and 70.8% of patients were aged between 6 and 59 months old [16]. The nutrition diagnosis made at the time [based on the weight-to-height ratio plotted on the local growth curve established by De Maeyer in 1959, the presence of nutritional edema, and serum albumin levels] [17] was reassessed using the ENA for SMART program, version October 2007, for standardization according to the WHO child growth standards [18]. Based on the WHO standards, only 84% of the children were classified as having SAM [16]. The others, classified as having moderate acute malnutrition or not suffering from AM, were excluded from subsequent analyses. All of the children hospitalized were treated according to the guidelines at that time [17].

During the follow-up of these subjects who became adults in the meantime, ie. 11 to 30 years after nutritional rehabilitation, 524 subjects from the initial cohort who were still living in the HZs of Miti-Murhesa and Katana were surveyed [16]. To assess long-term growth, these survivors (exposed) were compared to 407 unexposed subjects randomly-selected from the community [16].

An unexposed was defined as a subject who had no hospital history of SAM, had the same sex, was living in the same community, and was no more than 24 months older or younger than the exposed. We selected community unexposed randomly by spinning a bottle at the exposed adult’s home and enquiring door to door, starting from the nearest house towards which the bottle pointed [13]. Initially, we aimed to select one unexposed for each exposed. However, unexposed proved harder to recruit than exposed, as many feared the social stigma associated with childhood malnutrition. The selection of unexposed subjects was also limited by the number of eligible adults in the community [16].

For this study, only subjects aged more than 25 years and who had been admitted for SAM before the age of five years were considered. According to these two criteria, 145 former malnutrition sufferers were selected (exposed group). A total of 97 individuals aged more than 25 years, out of the initial 407, were selected as unexposed. All the respondents provided signed informed consent for participation in the study, either by written signature or by fingerprints, depending on literacy.

### Variables of interest

#### Dependent variables

The main variables of interest were clinical markers for HBP during adulthood. HBP was defined as a systolic BP (SBP) ≥ 140 mmHg and/or a diastolic BP (DBP) ≥ 90 mmHg and/or the taking BP-lowering drugs. The mean BP (MBP) and pulse pressure (PP) were calculated using the following formulas: MBP = DBP + 1/3(SBP - DBP), and PP = SBP - DBP [19].

#### Independent variables

For HBP, the main exposure was a history of SAM during childhood. Other variables such as occupational category, food consumption, sex and anthropometric measurements (BMI during adulthood) were taking into consideration when modelling for potential confounding factors. BMI (Body Mass Index) was calculated using the formula weight/height^2^ (in kg/m^2^) and BMI values were split into four categories: < 18.5 = underweight, 18.5 to 24.9 = normal, 25 to 29.9 = overweight and ≥ 30 = obese [20].

The type and frequency of food consumption were evaluated using a food consumption score created by the World Food Program [21, 22]. This score measures the dietary diversity of households, weighted according to frequency of consumption. We asked the head of the household (often the mother) how many days the household had eaten each of the ten following food groups in the last seven days: grains, tubers, legumes, vegetables, fruits, meat/fish, milk/dairy products, sugar, oil/fat, and condiments. The frequency with which each food group was consumed was then multiplied by its nutritional value, giving a score for each food group. Lastly, the scores for each food group were added to calculate the overall score. Depending on the total, a subject was considered as having an insufficient, borderline or satisfactory diet if his/her score was between 0 and 28; 28.5–42 and > 42 respectively [21, 22]. For salt consumption, each subject had to say whether they added dietary salt to food during meals. To measure the frequency of salt consumption, the participant chose one of the following responses: rarely, often or always.

The type of occupation was based on the “Classification Internationale Type des Professions”, the French version of the International Standard Classification of Occupations adapted to the European Union [23]. In order to apply it correctly in our context, we merged the occupational groups into group 1 (managers and administrative position), the second group included farmers, fishermen, market vendors and finally the rest of the groups were merged into group 3 (unskilled workers). The unemployed subjects were placed in group 3.

Smoking was taken into account for subjects who reported being occasional or regular smokers (more than 3 days per week) of at least one cigarette (including hand-rolled cigarettes), pipe or other form of tobacco [24]. Finally, we used the threshold of consuming at least 2 (women) or 3 (men) units of alcohol (beer or local alcoholic beverages, wines or spirits) per day, regularly (more than 3 times per week), for a subject to be recorded as a regular drinker of alcoholic beverages [25].

### Data collection

Data were collected over a period of five months (August 2018–December 2018), and the collection took place in two steps, carried out by 20 trained community health workers (CHW) and 2 supervisors, assisted by local leaders, qualified nurses and community liaisons. The CHWs were the same as those who had helped identify the subjects during the gathering of the cohort [16].

The first step consisted of a home visit. During these visits, the CHWs administered a sociodemographic questionnaire translated into Kiswahili (language spoken in the east of the DRC) to the participants, took their anthropometric measurements and scheduled an appointment within 48 h at the nearest hospital for the second step. This appointment involved taking a venous blood sample and BP measurements taken by properly trained nurses working in the various health facilities in the zone. Unlike the nurses and laboratory technicians who did not know whether a participant was an exposed or an unexposed, the CHWs knew this information.

The questionnaire covered variables relating to the participant’s identity, their lifestyle (alcohol and tobacco consumption, dietary habits), their medical history, known cardiovascular risk (CVR) factors (family or personal) as well as their socio-economic status (education and occupation).

The anthropometric measurements considered were weight and height. Body weight was measured to the nearest 100 g, with the subject wearing only light clothing, using electronic scales (bathroom scales, Tanita Digital HD-325®). Height was determined using a SECA 206 cm® measuring device with the subject wearing no shoes, to the nearest 0.1 cm. The anthropometric measurements were taken according to WHO guidelines [18] and were quality controlled, meaning that they were taken independently by two members of the team. The final measurement was the average of the two measurements. In the event of a discrepancy of more than 300 g (weight) and/or 0.5 cm (height), a third measurement was taken. The average of the two closest measurements was then used.

For all participants, BP was measured at rest, the subjects having abstained from strenuous physical activity, tobacco and/or alcohol consumption and beverages with a stimulating effect such as coffee and tea for a period. BP was measured using an electronic device (OMRON Hem 7001E®, Tokyo, Japan). Three measurements were taken with the left arm held at heart level, at five-minute intervals, with the subject seated and relaxed for at least 5 min. The cuff size was adjusted to the size of each participant’s arm. The average of the second and third BP measurements was used. If the SBP or DBP measurements differed by more than 10 mmHg, a fourth measurement was taken and the mean of the two closest measurements was used.

Lastly, 4 ml of blood was drawn via antecubital venipuncture after 12 h of fasting to determine serum creatinine levels using standard calorimetric enzymatic methods (CYAN smart CY009, Brussels, Belgium) at the laboratory of the general provincial referral hospital in Bukavu (tertiary hospital).

### Statistical analysis

We used Stata version 13.1 software for all analyses. Categorical variables were summarized in the form of frequency and proportion. Continuous data were presented as mean and standard deviation (SD).

The data from exposed and unexposed were compared using Chi-squared tests or Fisher’s exact tests (for proportion) and with Student’s t-tests (for means).

Linear and logistic regression models were used, respectively, for the continuous [creatinine levels and BP (systolic, diastolic, mean and pulse)] and dichotomous variables (HBP). The basic models only included the main exposure - SAM- providing an unadjusted difference in the means with a 95% confidence interval (95% CI) between exposed and unexposed for continuous variables and an unadjusted Odds Ratio (OR) for HBP.

Different models were then constructed in order to analyze the effects of SAM after adjustment. The adjusted model included three nominal variables accounting for the fixed effects on BP of occupational category, food consumption score and adult anthropometrics (BMI). The differences in the means and ORs are presented along with their 95% CI. In order to be included in the models, BMI, occupational category and food consumption score were converted into dichotomous variables. This is justified by two reasons: the small number of subjects in the subcategories of these different variables and the fact that for these different variables, it is classically documented that subjects with a high BMI, a good occupation or a satisfactory dietary situation would be the most at risk of cardiovascular diseases.

For occupation and food consumption score, we considered subjects with a satisfactory food consumption (food consumption score > 42) and satisfactory occupation (a management and administrative position for occupation) as positive responses. For BMI, since none of the thinness subjects developed hypertension, we combined this group with the normal BMI subjects to form the new class that served as a reference group in terms of BMI. We considered subjects with overweight and obesity as higher BMI. Other categories were considered as references (inadequate occupation, insufficient food consumption and normal BMI/underweight).

Conditions for applying a linear regression (normality, uniformity of variance and linearity) were verified via analysis of residuals, and goodness-of-fit was verified using the Hosmer and Lemeshow test.

## Results

We included a total of 145 subject exposed to SAM during childhood and 97 who were not exposed.

Table 1 shows the sociodemographic and economic characteristics of the study population.

**Table 1:** Sociodemographic and nutritional characteristics of the 2 groups

	Exposed			Unexposed			***p***-value
	N (total)	%	Mean (SD)	N (total)	%	Mean (SD)	
**Age (years)**	145		28.2 (3.4)	97		28.6 (3.5)	0.37
**Male**		57.2			56.7		0.51
**Civil status**							
Living alone		19.3			21.7		0.74
In a relationship		80.7			78.3		
**Ethnic group**							
Shi and Havu		99.3			97.9		0.15
Other ethnicities		0.7			2.1		
**Religion**							
Catholic		45.5			52.6		
Protestant		49.0			46.4		0.39
Other		5.5			1.0		
**Level of education**							
None		36.6			28.8		0.023
Primary		36.5			28.9		
Secondary		24.8			33.0		
University		2.1			9.3		
**Occupational category**							
Managerial and administrative position		4.1			14.4		0.011
Farmer + fishermen + market vendor		62.8			61.9		
Unskilled worker		33.1			23.7		
**Nutritional survey**							
**1. Food consumption score**							
Inadequate		11.7			11.3		0.79
Borderline		40.0			36.1		
Satisfactory		48.3			52.6		
**2. Addition of salt to meals**							
Rarely		74.5			81.4		0.23
Often		22.7			14.4		
Always		2.8			4.1		

The mean age and the ratio of men to women were similar for the two groups. Compared to unexposed, exposed had lower education level and lower skilled occupations. These differences were statistically significant. We did not however observe a significant difference in civil status, ethnic group, religious affiliation, or food and salt consumption between the two groups.

Tables 2 and 3 show the clinical and biological markers and the prevalence of the different risk factors for NCDs in the two groups.

**Table 2:** Prevalence of HBP risk factors among the two groups

	Exposed			Unexposed		
	N (total)	%	Mean (SD)	N (total)	%	Mean (SD)*
**Anthropometry**	145			97		
Height (cm)			158.1 (8.2)			157.7 (8.6)
Weight (kg)			55.2 (7.9)			55.9 (7.2)
BMI° (kg/m^2^)			22.1 (2.7)			22.5 (2.6)
< 18.5		4.8			4.1	
18.5–24.9		80.7			79.4	
25.0–29.9		13.8			15.5	
> 30.0		0.7			1.0	
**Cardiovascular risk factors**						
1. Alcohol (yes)		49.0			49.5	
2. Tobacco (yes)		4.8			5.2	
3. Family history of HBP^1^ and/or diabetes		35.2			36.1	

*For all of the variables, no statistically significant difference was observed between the two groups

^1^HBP=High Blood Pressure. °BMI=Body Mass Index

**Table 3:** Clinical and biological markers and prevalence of HBP in the two groups

	Exposed			Unexposed		
	N (total)	%	Mean (SD)	N (total)	%	Mean (SD)*
	145			97		
**Blood pressure (BP) mm Hg**						
Systolic BP			120.4 (12.1)			120.1 (12.9)
< 120		50.3			44.3	
120–139		42.1			49.5	
≥ 140		7.6			6.2	
Diastolic BP			73.5 (10.3)			75.1 (9.2)
< 80		73.1			68.0	
80–89		19.3			23.7	
≥ 90		7.6			8.3	
Mean BP			89.1 (9.6)			90.0 (9.3)
Pulse pressure			46.9 (10.9)			44.9 (10.6)
**High blood pressure**		7.6			4.1	
**Serum creatinine (mg/dL)**			0.91 (0.2)			0.95 (0.2)

*For all of the variables, no statistically significant difference was observed between exposed and unexposed

Looking at NCD risk factors, none of the studied variable was significantly different between the two groups (Table 2).

Although overall low, HBP prevalence was clearly higher among exposed than among unexposed. Exposed subjects had a mean PP higher than that of unexposed, by the contrast, their DBP was lower than unexposed. However, the differences observed were not statistically significant. In addition, MBP, SBP and serum creatinine levels were similar between groups (Table 3).

Table 4 shows the unadjusted and adjusted differences in HBP markers between the two groups.

**Table 4:** Difference in Blood Pressure and serum creatinine level between exposed and unexposed

	Unadjusted difference (95% CI) N*	***p***-value	Adjusted difference (95% CI)	***p***-value
**Blood pressure (BP) mm Hg**		0.837		
Systolic BP	0.4 (−3.2, 4.0)		0.7 (−2.9, 4.2)	0.72
Diastolic BP	−1.4 (−4.4, 1.4)	0.294	−1.5 (−4.5, 1.4)	0.31
Mean BP	−0.9 (−3.7, 1.9)	0.523	−0.6 (− 3.6, 1.9)	0.57
Pulse Pressure	1.9 (−1.2, 5.1)	0.234	2.2 (−1.1, 5.4)	0.18
**Creatinine (mg/dL)**	−0.04 (− 0.07, 0.01)	0.153	− 0.04 (− 0.09, 0.01)	0.12

Adjusted for BMI, occupational category and food consumption

*N = 145 cases and 97 controls

The models show that after adjusting for BMI, food consumption score and occupation, the differences in SBP, DBP and PP between exposed and unexposed increased, without reaching the threshold of statistical significance, while the difference between the MBP decreased.

Table 5 shows a summary of the major independent predictors of HBP among the population.

**Table 5:** Independent predictors of High Blood Pressure among the total population of the study

	Total Population	HTA	Unadjusted OR (95% CI)	***p***-value	Adjusted OR (95% CI)	***p***-value
	N	yes	***N*** = 15			
**BMI**						
Normal	205	8	Ref		Ref	
Higher BMI	37	7	5.7 (1.1, 8.4)	0.012	5.3 (1.1, 8.6)	0.002
**Occupation**						
Inadequate	222	12	Ref		Ref	
Satisfactory	20	3	3.1 (1.3, 16.3)	0.015	3.8 (0.9, 16.3)	0.069
**Food consumption score**						
Insufficient	121	5	Ref		Ref	
Satisfactory	121	10	2.1 (0.8, 6.3)	0.10	1.7 (0.5, 5.2)	0.31
**Sexe**						
Female	104	2	Ref		Ref	
Male	138	13	5.3 (1.6, 31.7)	0.010	11.2 (2.2, 55.7)	0.003
**SAM in childhood**						
Non exposed	97	4	Ref		Ref	
Exposed	145	11	1.9 (0.7, 5.6)	0.21	3.1 (0.9, 10.9)	0.064

After adjustment, the variables significantly associated with HBP were being male and having higher BMI. Exposure to SAM and a satisfactory occupation were associated with a higher risk of HBP, did not reach statistical significance. A satisfactory food consumption score was not associated with HBP.

## Discussion

Our aim was to analyze the potential association between an episode of SAM occurring before the age of 5 years and risk of raised BP in adulthood in an environment without nutrition transition.

Our results suggest that mean BP values measured were comparable between subjects who experienced SAM during childhood and those who did not. The prevalence of (known) HBP was almost twice as high in subjects who were exposed to SAM during childhood compared to unexposed subjects. In addition, the risk of developing HBP during adulthood tended to be higher among exposed than unexposed. This study indicates that being male and having a higher BMI were the main independent contributors to HBP among this still relatively young population.

To our knowledge, this study is the first from sub-Saharan Africa to assess the long-term effects of childhood SAM on BP variability in young adults after a monitoring period ranging from 11 to 30 years, after discharge from hospital, in a context of endemic malnutrition. Our study is original in several respects: it examined major contributors to BP, included subjects who continued to live in an unfavorable environment without nutrition transition, and its analysis considered several CVR factors including lifestyles (alcohol, tobacco and food consumption) and occupational category as a proxy of socio-economic status.

As regards renal function, serum creatinine levels were similar between the two groups. This lack of difference may result from the fact that kidney development mostly occurs during the intra-uterine period, with no new nephrons formed after birth [26]. Therefore, in contrast to intra-uterine undernutrition, which leads to reduced nephrotic capital in survivors and which may explain the long-term hemodynamic modifications and nephrocalcinosis involved in the genesis of HBP and chronic renal diseases in adulthood [26], our subjects already had a stable nephrotic capital that was less sensitive to SAM. This would also partly explain the lack of difference in BP variability between the two groups. We did not observe any statistically significant difference between the two groups in terms of SBP, DBP, PP or MBP.

Our study shows that HBP prevalence was almost twice as high among those exposed to SAM than among unexposed. This result corroborates a study conducted in Nigeria that showed a higher prevalence of HBP in participants born during the Nigerian Civil War and who had been exposed to severe famine both in utero and during childhood, compared to control subjects who lived in the same region before th1965–1967 civil war [15].

The exposed presented an increased risk of developing HBP during adulthood, despite the difference observed being not statistically significant, probably due to the small sample size of the study. The results of our work are consistent with those of a study conducted in Malawi on pre-pubescent children [13]. This study in Malawi identified children exposed to SAM as not being at greater risk of developing HBP than children not exposed to SAM. Our results are also comparable to those of a study in rural Uganda which identified factors associated with recovery from undernutrition as being more significant with respect to the development of HBP during adolescence than undernutrition itself [14]. In our study, the subjects lived in a context characterized by an absence of nutrition transition, with precarious socio-economic conditions persisting during and after recovery from SAM.

Nevertheless, our results differ from almost all studies conducted in HICs, which show that subjects with low birth weight and/or low weight gain in childhood were at statistically significant higher risk of developing HBP and/or having higher BP during adulthood than controls [27–29]. This discrepancy may be caused by several factors, including different ethnicities, since our subjects were all sub-Saharan Africans whereas the vast majority of HICs inhabitants are Caucasians. These ethnicity-related differences could be attributed in part to genetic [30] and environmental factors. As such, sub-Saharan populations are characterized as having different determinants for the development of HBP and metabolic handling of normal or excess salt intakes, which could have a confounding effect on the data observed.

Secondly, there were major differences in age between the populations. The majority of our subjects were young adults (mean age 28 years), unlike those in studies from HICs (median age 50 years) [28, 29, 31, 32]. This would also partly explain the absence of effect of age on BP variability and the development of HBP, given that the effects of natural ageing on BP and HBP become more apparent after the age of 50 years [27]. As our population was still relatively young, and given that the risk of NCDs increases with age, an additional 10–20 years of hindsight would be needed to likely increase HBP prevalence in this population.

Thirdly, lifestyle and socio-economic status before and after exposure to the episode of undernutrition differ between the two regions. In contrast to the studies conducted in HICs, our subjects spent their childhood in precarious nutritional conditions before experiencing one or more episodes of SAM, and then continued to live in an unfavorable environment in terms of food quality and security, without nutrition transition. In HICs, famines occurred in populations that generally had a high socio-economic status and good health prior to the episode of famine, and rapidly recovered this status afterwards [28, 29, 33], whereas our cohort remained relatively disadvantaged during and after the episode of SAM, and therefore unexposed to an obesogenic environment up to adulthood. As a rule, people in South Kivu have little access to processed and/or industrialized food. The population keeps on consuming local foods, with reduced fat content and poor in refined carbohydrates. However, one cannot rule out that target organs damage could become more apparent once they are subsequently exposed over long periods to Western-style lifestyles promoting weight gain.

The fourth reason could be the life history period of exposure to undernutrition. Our subjects were exposed to SAM during childhood and not in utero, as was the case in the majority of the HICs. In contrast to changes to organ structure and function during the intra-uterine period, which are only partially reversible [34], the majority of organs already reached full developmental maturity during the childhood, and the changes associated with SAM could be less permanent than those occurring during the rapid fetal growth period, reducing the long-term effects of childhood SAM compared with fetal malnutrition.

Lastly, the difference between the criteria for diagnosing undernutrition must be taken into consideration. In HICs, undernutrition was defined based on a reduction in weight gain whereas, in our population, undernutrition was defined on the basis of weight-to-height ratio, mid-upper arm circumference and/or the presence of nutritional edema. In addition, more than 90% of our subjects had delayed growth during childhood [16]. Consequently, the effect of weight gain could be different in children who gained weight and height in a balanced way compared to those who gained weight and BMI, but had delayed growth. All of these factors may explain the differences in the findings observed.

We observed that only a higher BMI (overweight/obesity) and being male were major independent predictors of HBP in adulthood among our population. Our results corroborate those of other studies [34, 35]. BP is known to increase alongside BMI. In addition, being male is a well-documented unmodifiable CVR factor [35–37].

There are clear limitations of this study. Firstly, there is the survival bias: only those subjects who survived to adulthood and were still present in the villages two decades after the episode of SAM were studied. There is, however, no obvious reason to consider that the association between nutritional state of children admitted for SAM and BP would be different among those lost to follow up, due to the fact the hospital admission characteristics did not differ between subjects lost to follow up and subjects included [16].

Secondly, our study sample was not very large. Certain differences might have reached statistical significance with a larger sample size.

Thirdly, we did not have data on birth weight and height, gestational age, and on whether individuals were born as twins, triplets, etc., or on rate of growth in the first years of life. These factors could be potential confounders as they are linked to both SAM and to unfavorable late-onset phenotypes in terms of NCDs [38].

Fourthly, although not malnourished in the past, unexposed lived in the same unfavorable conditions as exposed, and it is difficult to establish whether they were perfectly healthy on a cardiometabolic level. The continued unfavorable situation in which our two groups lived probably helped significantly reducing any differences in most cardiometabolic markers studied.

Finally, in our study, BP was only measured on one day, without subsequent control measurements. This could have altered the differences observed, in one direction or another.

## Conclusion

Our results show that an episode of SAM in childhood has a weak impact on BP variability in young Congolese adults (from DRC) living in an environment without nutrition transition. However, subjects who experienced a period of SAM during childhood had a higher prevalence of HBP and a moderately higher risk of developing HBP than unexposed subjects. Multicentre studies involving larger cohorts of older adults would provide greater understanding of the impact of SAM on the overall risk of CVDs and BP disorders in adulthood. Finally, our results are sufficient to show that efforts to combat SAM and its consequences should remain a public health priority. Given the paradoxical of high prevalence of SAM in LICs and lack of scientific data on the potential long-term consequences of SAM, our results provide useful insights in this field.

## Data Availability

All data generated or analyzed during this study are included in this published article.

## Abbreviations

AM: Acute malnutrition
BMI: Body Mass Index
BP: Blood Pressure
CHW: Community Health Workers
CM: Chronic Malnutrition
CRSN-Lwiro: Centre de Recherche en Sciences Naturelles de Lwiro
CVDs: Cardiovascular Diseases
CVR: Cardiovascular Risk
DBP: Diastolic Blood Pressure
DRC: Democratic Republic of the Congo
HBP: High Blood Pressure
HMICs: High-Middle Income Countries
HPL: Lwiro Pediatric Hospital
HZ: Health Zones
LICs: Low-Income Countries
MBP: Mean Blood Pressure
NCDs: Non-Communicable chronic Diseases
OR: Odds Ratio
PP: Pulse Pressure
SAM: Severe Acute Malnutrition
SBP: Systolic Blood Pressure
WHO: World Health Organization
